# Rare giant malignant tumors of the chest - reconstructive challenge

**DOI:** 10.1186/1749-8090-10-S1-A116

**Published:** 2015-12-16

**Authors:** K Kurowski, Corcoles JM Padilla, JI Matuszek

**Affiliations:** 1 Department of Thoracic Surgery, Hospital Universitario del Vinalopo, 03293,Elche, Alicante, Spain; 2 Department of Anesthesiology, Hospital Universitario de Torrevieja, 03186, Torrevieja, Alicante, Spain

## Background/Introduction

In this article, we would like to present an unusual primary malignant tumors of the thorax. Case history and radiological studies of 4 patients with histologic diagnosis of thoracic sarcomas were interpreted retrospectively.

## Aims/Objectives

Tumors originated from the chest wall, mediastinum, and pulmonary parenchyma was included. Histopathologic diagnoses were: thymo-liposarcoma, leiomyosarcoma, malignant Schwannoma and malignant mesothelioma - sarcomatoid type. In order to evaluate thoracic sarcomas, cross-sectional methods such as CT and MRI can be useful in demonstrating the origin of the mass, relationship with and involvement of adjacent structures. Chest wall resections are associated with significant morbidity, with respiratory failure in as many as 27% of patients. We hypothesized that our selective use of Dual-mesh prosthesis and STRATOS System (Strasbourg Thoracic Osteosyntheses) for chest wall reconstruction reduces respiratory complications.

## Method

The records of all patients with giant tumors undergoing chest wall resection and reconstruction were reviewed. Patient demographics, use of preoperative therapy, the location and size of the chest wall defect, performance of lung resection if any, the type of prosthesis, and postoperative complications were recorded.

## Results

From February 2009 to January 2014, 4 patients underwent surgical treatment and chest wall resection for giant chest tumor. The median defect size was 80 cm2 and the median number of ribs resected was 3. Lung resection was performed in 3. Prosthetic reconstruction was done with use of dual and polypropylene mesh and STRATOS system. Postoperatively, 1 patients died (had pneumonectomy plus chest wall resection. Respiratory failure did not occurred.

## Discussion/Conclusion

The use of malleable Titanium bars to restore anatomic rib continuity helps to preserve the mechanics of ventilation better than a soft patch repair.

We have no incidence of respiratory failure. Pneumonectomy plus chest wall resection should be performed only in highly selected patients.

